# Turning on fluorescent probe for sensitive detection of streptomycin in pure, pharmaceutical formulations and human plasma

**DOI:** 10.1186/s13065-025-01708-7

**Published:** 2026-01-17

**Authors:** Bassant Samy, Mokhtar M. Mabrouk, Mohamed A. Abdel Hamid, Hytham M. Ahmed

**Affiliations:** 1 Pharmaceutical Analysis Department, Faculty of Pharmacy, Menoufia University, Shebin Elkom, Menoufia Egypt; 2 Department of Pharmaceutical Analytical Chemistry, Faculty of Pharmacy, Tanta University, Tanta, El Gharbeia Egypt; 3 Department of Pharmaceutical Chemistry, Faculty of Pharmacy, Alsalam University, Kafr El Zayat, El Gharbeia Egypt; 4 Pharmaceutical Analytical Chemistry Department, Faculty of Pharmacy, Menoufia National University, 70 km Cairo-Alexandria agricultural road, Menoufia, Egypt

**Keywords:** Streptomycin, Fluorescamine, Coplanar structure, Spectrofluorimetry, Human plasma

## Abstract

Streptomycin is a clinically important aminoglycoside antibiotic widely used for treating tuberculosis and other bacterial infections. Accurate determination of its concentration is essential for quality control, therapeutic drug monitoring, and preventing toxic effects associated with overdosing or accumulation in the environment. In this work, a simple, rapid, and highly sensitive spectrofluorimetric method was developed for quantifying streptomycin in pure form, pharmaceutical vials, and human plasma using fluorescamine as a fluorogenic probe. The method is based on the reaction of fluorescamine with the primary amine group of streptomycin under mild alkaline conditions to yield a fluorescent product emitting at 482 nm (excitation 390 nm). The calibration curve was linear over the range 100–600 ng/mL, with a correlation coefficient of 0.9999, a detection limit of 8.3 ng/mL, and a quantitation limit of 25.2 ng/mL, ensuring sensitive and accurate determination across the studied range. The procedure demonstrated high precision (RSD < 2%) and accuracy (recoveries 98.1–101.3%). The method was successfully applied to commercial streptomycin vials (% recovery = 99.7 ± 0.8) and to spiked human plasma samples with negligible matrix interference. Owing to its simplicity, short analysis time (3 min), and excellent reproducibility, the proposed assay offers a cost-effective alternative to chromatographic methods for routine quality control and bioanalytical applications.

**Graphical abstract:**

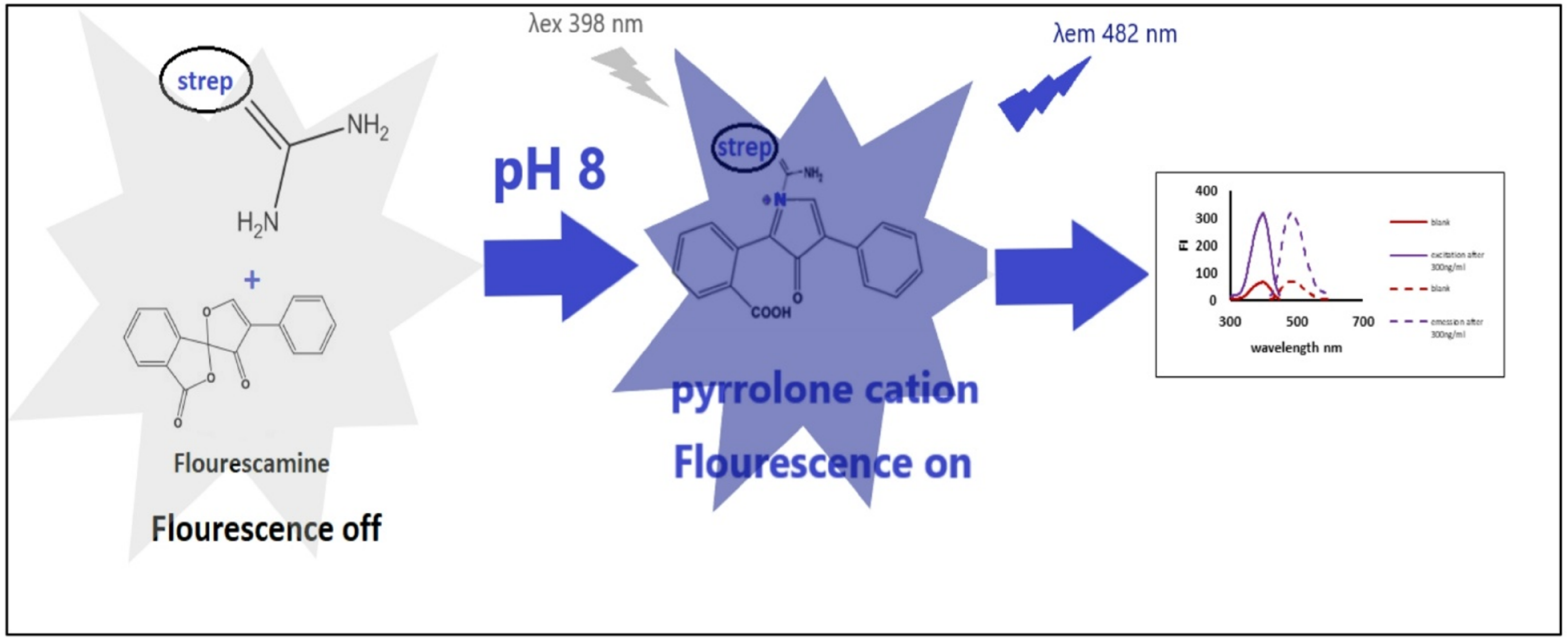

**Supplementary Information:**

The online version contains supplementary material available at 10.1186/s13065-025-01708-7.

## Introduction

Streptomycin is an aminoglycoside antibiotic exhibiting broad-spectrum targeting Gram-negative and Gram-positive bacteria. Streptomycin efficacy in conjunction with other drugs in treatment of tuberculosis is one of its biggest assets [1, 2]. Kidney and ear damage are the most serious adverse effects, like with other aminoglycosides. As a result, serum levels must be measured for therapeutic and toxicological purposes [3]. Due to chemical structure of Streptomycin (Fig. S1) which lacks chromophore or fluorophore groups in its molecule, direct UV or fluorimetric detection is ineffective. So, the chemical derivatization of primary amino groups is frequently used [4]. For the analysis of the examined cited drug, many analytical methods have been presented, including: spectrophotometry [5–10], high performed liquid chromatography (HPLC) [11–13], capillary zone electrophoresis [14], spectrofluorometric [15–17]. Some of these previous experiments need costly equipment, time-consuming. Also, pre-treatment steps such as heating that are difficult to come by in regular laboratories, or some of these methods are insensitive. Spectrofluorimetry is a well-known technique that is sensitive and used for quantitative analysis [18–21]. Fluorescamine is a well-known fluorogenic reagent that reacts rapidly with primary aliphatic amines to form a highly fluorescent pyrrolone derivative, a mechanism that was extensively characterized upon its discovery [22–23]. Although the fact that fluorescamine is non-fluorescent itself, it rapidly interacts with primary amino groups to produce extremely luminous compounds. Fluorescamine is preferable rather than other fluorogenic agents due to its simplicity, fastness, and no need for heating. Recently, advanced methods have continued to emerge. Highly sensitive chromatographic techniques like hydrophilic interaction liquid chromatography tandem mass spectrometry (HILIC-HPLC-MS/MS) can achieve detection limits as low as 0.006 ng/mL for environmental analysis [24], and LC-MS/MS methods are routinely applied to biological matrices like plasma [25]. While these are powerful confirmatory techniques, their high cost and operational complexity limit their use in routine labs. Concurrently, modern spectrofluorimetric methods utilizing nanomaterials such as carbon quantum dots [26] and silver nanoparticles [27] have been developed, offering good sensitivity and selectivity. However, these often involve time-consuming and complex probe synthesis. Therefore, there remains a clear need for a straightforward, rapid, and cost-effective method that does not sacrifice sensitivity or reliability for the routine determination of streptomycin in pharmaceutical and clinical samples. This work aims to fill this gap by developing a validated spectrofluorimetric method based on the simple derivatization of streptomycin with fluorescamine. The primary amino group of streptomycin enables activation of fluorescamine fluorescence in a buffered medium at pH 8, with emission recorded at 482 nm upon excitation at 390 nm. The developed quantitative method is suitable for application in quality control laboratories.

## Experiment

### Instruments

Fluorescence spectrums were analyzed by a JASCO model FP 6300 spectrofluorimeter with a xenon lamp & 1 cm cells made of quartz. It is connected with the spectra manager device. The spectrofluorometric measurements were conducted using a slit width of 10 nm and a scanning speed of 1000 nm/min. The pH values of the solutions were determined with an Adwa pH meter (Model AD1030, Romania), while sample weighing was performed using a high-precision analytical digital balance (Switzerland).

### Materials and reagents

All chemicals and reagents employed were of analytical grade. Pure streptomycin (Strep., declared purity 99.8% by the supplier) was kindly provided by the National Organization for Drug Control and Research (NODCAR), Giza, Egypt. Commercially available Streptomycin^®^ vials containing 1 g of streptomycin sulfate were purchased from a local pharmacy. Fluorescamine (≥ 99% purity, Sigma-Aldrich, Germany) was freshly prepared as a 0.04% (w/v) solution in acetone prior to use. Borate buffer (pH 7.5-9) was made by combining 0.1 M boric acid and 0.1 M sodium hydroxide (NaOH, GIO-Chem, 99% purity) in a 1:1 ratio using a pH meter to adjust the pH to the desired range. Human plasma samples were generously supplied by the Blood Bank of Menoufia University Hospitals (Menoufia, Egypt). The samples were stored at − 20 °C until analysis and thawed immediately before use in a 38 °C water bath with gentle agitation.

### Standard solutions

Stock solution of Streptomycin (0.1 mg /mL) prepared via dissolving 10 mg of Streptomycin in distilled water in a 100 mL volumetric flask. Working solutions were prepared by serial dilution of the stock solution using the same solvent. To achieve a required final concentration (100–600 ng/mL).

### General analytical procedures

Aliquots of streptomycin working solutions, covering the desired concentration range, were transferred into a series of calibrated 10 mL volumetric flasks. To each flask, 1.0 mL of borate buffer (pH 8.0) and 0.75 mL of fluorescamine solution (0.04% w/v) were added, and the volume was adjusted to the mark with distilled water. The mixtures were left to stand for 3 min to allow complete reaction, fluorescence was then measured at 482 nm following excitation at 390 nm. A blank was prepared via the identical conditions except that the drug solution was omitted which used for comparison.

### Procedure for the commercial vials

Five vials of Streptomycin^®^ were mixed then, an accurately weighed 10 mg of Streptomycin was placed into a 50 mL volumetric flask. Complete the flask to the mark with water. Then transfer accurately 2.5mL of this solution to 100mL calibrated flask to obtain a final concentration of 5 µg/mL and proceed as general analytical procedure.

### Procedure applied to spiked human plasma

Add quantitively 100 µL of Streptomycin standard stock solution to 1000 µl of plasma in a centrifuge tube. Then mixed well with a vortex mixer, after that added 3 mL of acetonitrile to the tube as precipitating agent. The resulted mixture was centrifuged for 20 min at 5000 rpm. To determine the working concentration range, different volumes of the supernatant were withdrawn. After that, the general analytical procedures were followed.

## Result and discussion

**Fig. 1:**
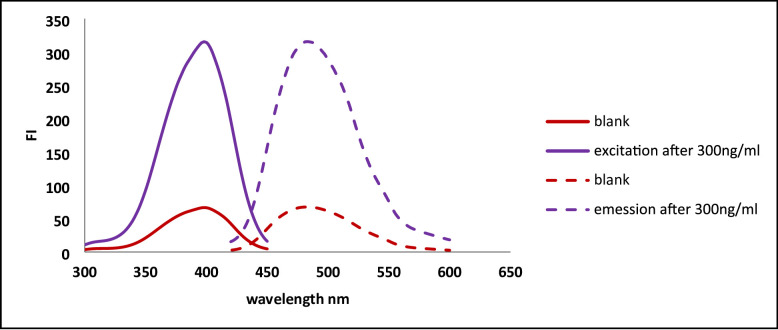
The excitation and emission spectra of the blank and the reaction product between Streptomycin (300ng/mL) and fluorescamine

**Fig. 2:**
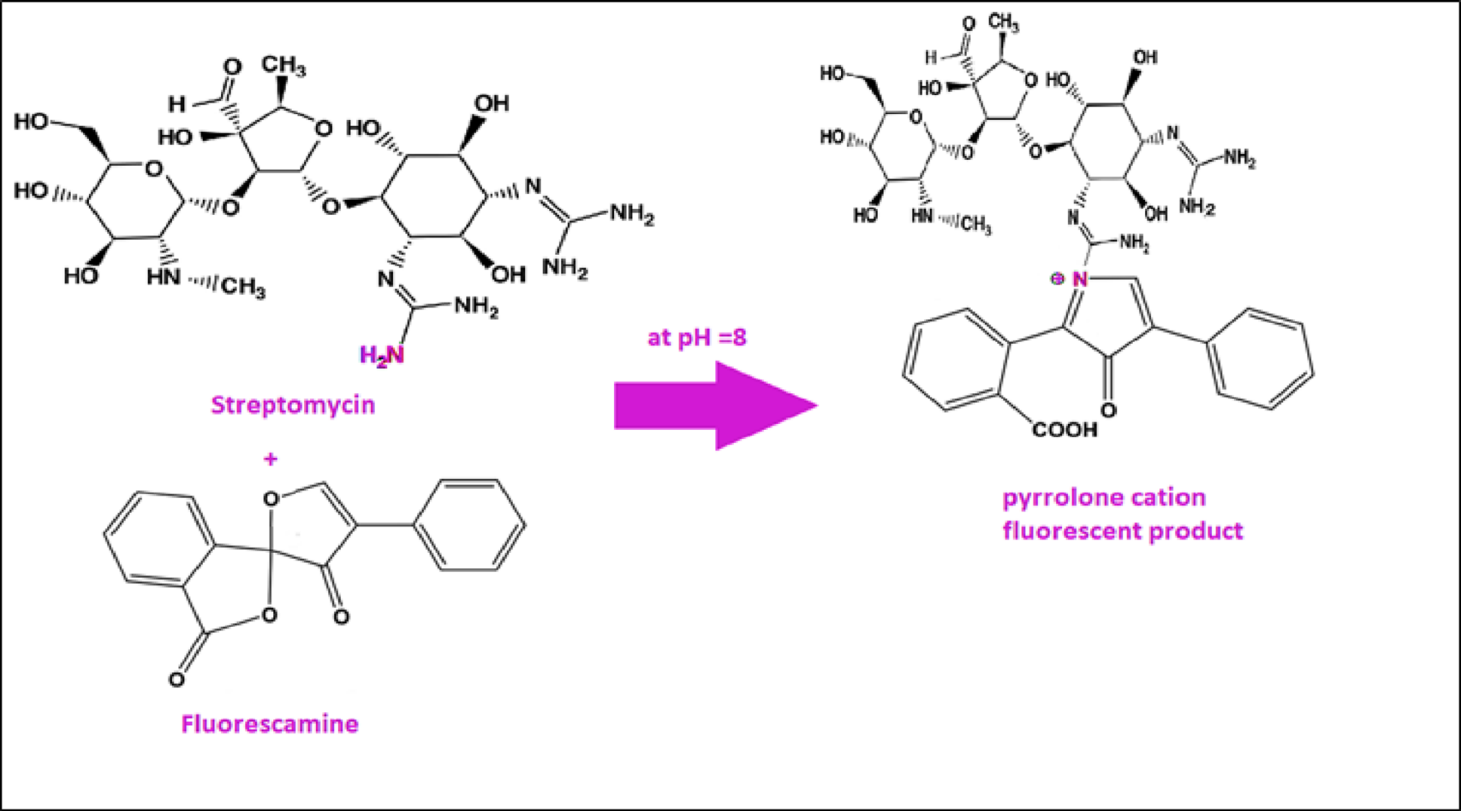
The suggested chemical reaction for the fluorescence switch on the reagent by the primary amino group of Streptomycin

Fluorescamine is extensively utilized as a fluorogenic reagent for the analysis of pharmaceutical agents bearing primary amines [22–23]. Fluorescamine is preferable rather than other fluorogenic agents due to its simplicity, fastness, and no need for heating. Fluorescamine itself is non-fluorescent; however, upon reacting with amino groups, it yields a highly fluorescent pyrrolone cation. In alkaline medium, the pyrrolone cation exists as an unsaturated, conjugated, and planar structure with high rigidity, which accounts for its strong fluorescence. While under acidic or highly alkaline conditions fluorescamine forms a non-planar, less conjugated derivatives [28–29]. The fluorescence of fluorescamine is initiated through its reaction with streptomycin’s primary aliphatic amine group, producing a product that emits at 482 nm upon excitation at 390 nm (Fig. 1). The proposed reaction mechanism is illustrated in Fig. 2.

### Optimization of experimental variables

Experimental variables such as pH, buffer volume, reaction time, and fluorescamine volume were optimized using a one-factor-at-a-time (OFAT) approach where each variable was altered while the other remained unchanged. Streptomycin was used at a final concentration of 300 ng/mL in all experiments.

#### The pH effect

**Fig. 3:**
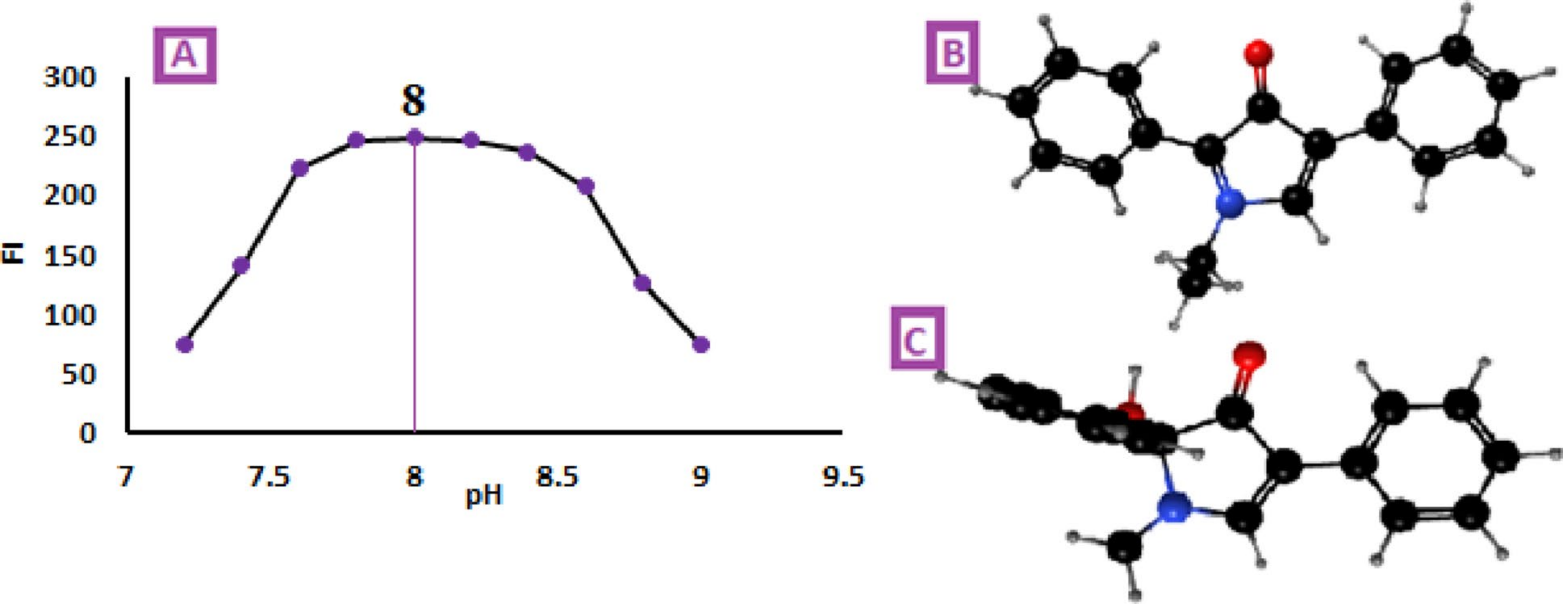
**A** Effect of pH on FI of the reaction product between Streptomycin (300ng /mL) and fluorescamine reagent **B** the 3D structure of pyrrolone cation (planar) at pH = 8 **C** the 3D structure of hydroxylated pyrrolone (non-planar) at highly alkaline medium

This pH-dependent profile is a characteristic signature of the fluorescamine reaction mechanism [22, 23]. Fluorescence was observed exclusively under alkaline conditions, whereas it disappeared completely in acidic or strongly alkaline media because of formation of a non-planar derivative. Accordingly, the pH range for the study was restricted to 7.5–9. As illustrated in Fig. 3, the fluorescence intensity (Fl) increased progressively, reaching a maximum within the pH range of 7.6–8.6, and then declined at higher pH values. This decrease is attributed to the formation of hydroxylated pyrrolone, a non-planar species with reduced conjugation compared to the cationic pyrrolone, which possesses a more rigid, planar structure. The optimum pH for the reaction was determined to be 8.0 ± 0.2, as it yielded the maximum fluorescence intensity (Fig. 3A). This pH is ideal for stabilizing the highly fluorescent, planar pyrrolone cation formed from the reaction of fluorescamine with primary amines, while avoiding the formation of the non-fluorescent hydroxylated pyrrolone that predominates in strongly alkaline conditions [22, 23, 29].

#### Effect of buffer volume

**Fig. 4:**
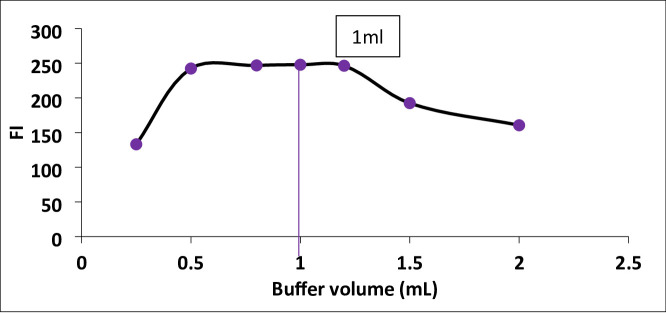
Effect of buffer volume on FI of the reaction product between Streptomycin (300 ng/mL) and fluorescamine reagent

The influence of buffer volume on the intensity of fluorescence was evaluated by varying the borate buffer volumes (pH 8). As shown in Fig. 4, optimum fluorescence was achieved at 1.0 ± 0.2 mL. Therefore, a volume of 1.0 mL of borate buffer (pH 8) was selected as optimal, as it provided the necessary buffering capacity for consistent pH without causing dilution-related quenching of the fluorescence signal (Fig. 4).

#### Effect of fluorescamine volume

**Fig. 5:**
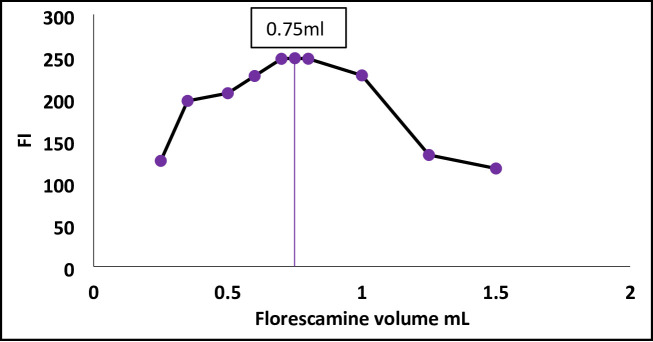
Effect of fluorescamine volume on FI of the reaction product between Streptomycin (300ng /mL) and fluorescamine reagent

Variation in fluorescamine concentration was found to impact the fluorescence response of the reaction product. Fluorescamine solution (0.04% w/v) was used in various volumes. Figure 5 shows that the fluorescence intensity increased as the reagent volume was increased till 0.8 mL, then decreased. A volume of 0.75 mL was selected for all subsequent experiments as it represented the optimal balance, providing a sufficient reagent excess for complete reaction without causing a decrease in fluorescence intensity, which can occur at very high concentrations due to inner-filter effects or self-quenching (Fig. 5).

#### Effect of reaction time

**Fig. 6:**
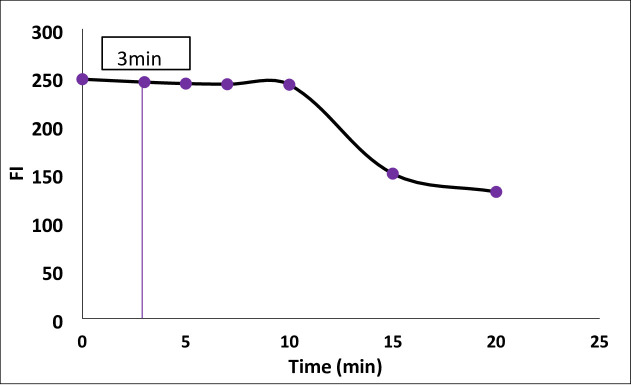
Effect of reaction time on FI of the reaction product between Streptomycin ( 300 ng /mL) and fluorescamine reagent

As shown in Fig. 6, fluorescamine interacted instantaneously with Streptomycin, and the intensity of fluorescence was unaffected by a standing time of up to 10 min. As a result, the study was completed in 3 min.

### The stoichiometry of the reaction

**Fig. 7:**
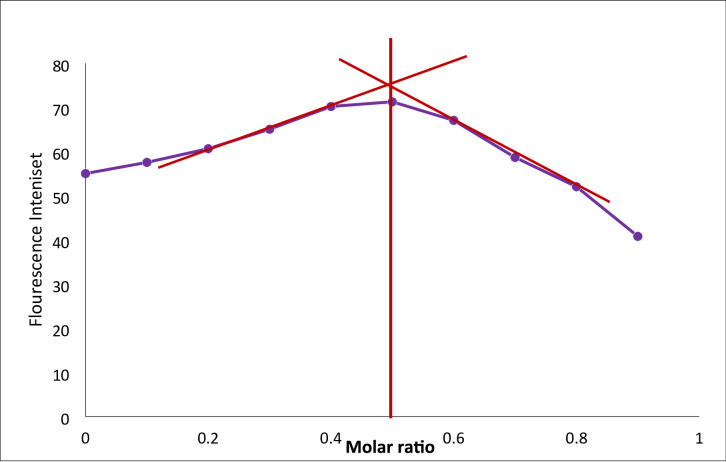
Job’s method for determination of the stoichiometry of the reaction of both Streptomycin and fluorescamine

Stoichiometric relationship of the Reaction was elucidated using Job’s method of continuous variation. It was noted that Streptomycin and fluorescamine have a 1: 1 molar ratio as presented in Fig. 7, confirming the postulated mechanism showed in Fig. 2, confirming that a single primary amine group on the streptomycin molecule reacts with one molecule of fluorescamine.

### Validation of the suggested methodology

The proposed method was validated following the key principles of bioanalytical method validation as outlined in regulatory guidelines (e.g., FDA Bioanalytical Method Validation Guidance) [30] to assist of the limit of detection (LOD), limit of quantitation (LOQ), as well as accuracy, precision, and robustness.

#### Linearity & range

A series of streptomycin standard solutions at different concentrations were analyzed using the established procedure. By plotting the obtained Fl against the corresponding Streptomycin concentration, the calibration curve was created. Statistical parameters of the calibration curve are presented in Table 1. A linear relationship was established between streptomycin concentration and fluorescence intensity covering the range of 100–600 ng/mL, exhibiting excellent linearity with a correlation coefficient (r²) of 0.9998.

**Table 1:** Statistical data and regression parameters for streptomycin determination by spectrofluoeimtery for the proposed method

Parameter	Streptomycin
λex (nm)	398
λem (nm)	482
Linearity Range (ng/mL)	100–600
Intercept (a)	174.328
SD of Intercept (Sa)	0.624
Slope (b)	0.247
SD of Slope (Sb)	0.001
Correlation coefficient (r)	0.9999
Coefficient of determination (r^2^)	0.9998
SD of residual (Sy/x)	0.671
Limit of detection (ng/mL)	8.3
Limit of quantitation (ng/mL)	25.2

^a^ standard deviation

#### Limits of detection (LOD) and quantitation (LOQ)

Method sensitivity was assessed based on the limits of detection (LOD) and quantitation (LOQ). The LOD and LOQ were calculated using the equations: LOD = 3.3σ/S and LOQ = 10σ/S, where *S* represents the slope of the calibration curve and *σ* denotes the standard deviation of the intercept. Based on these calculations, the LOD and LOQ were determined to be 8.3 ng/mL and 25.2 ng/mL, respectively.

When compared with previously reported spectrophotometric and spectrofluorimetric methods [5, 15–17], these values confirm the superior sensitivity of the developed method. A more comprehensive comparison with the latest reported methods is provided in Table 2. This comparison reveals that the sensitivity of the proposed method is competitive with modern techniques. For instance, it is more sensitive than some recent LC-MS/MS methods applied to biological tissues [25] and comparable to advanced nanomaterial-based fluorimetric probes [26, 27], while being significantly less sensitive than specialized HILIC-HPLC-MS/MS techniques used for trace environmental analysis [24].

**Table 2:** Comparison of the proposed spectrofluorimetric method with other reported methods for the determination of streptomycin

Method / principle or reagent	Reaction Conditions (Temp/time)	LOD	Sample Type	Key Advantages / Limitations	Ref.
Proposed Method Spectrofluorimetry (Derivatization with Fluorescamine)	25 °C 3 min	0.008 µg/mL	Pure, Pharmaceutical, Human Plasma	Adv: Simple, rapid, room temperature, cost-effective, high recovery in plasma. Lim: Derivatization required.	This work
Spectrophotometry (Potassium iodide and iodate)	25 30 min	0.011 µg/mL	veterinary dosage forms	Adv: - Lim: Moderate sensitivity, longer reaction time.	[5]
Spectrofluorimetry (9,10-Phenanthraquinone)	25 30 min	0.006 µg/mL	Pharmaceutical, Plasma	Adv: Sensitive. Lim: Longer reaction time	[15]
Spectrophotometry (Ninhydrin)	90–100 45 min	0.1 µg/mL	Pharmaceutical, Plasma	Adv: - Lim: Requires heating, lengthy procedure	[16]
Spectrophotometry (2,4-dinitrophenol)	25 10 min	3.284 µg/mL	Pharmaceutical	Adv: Simple. Lim: Low sensitivity.	[17]
Chromatography (HPLC-MS/MS)	-	0.001 µg/mL	Milk	Adv: High sensitivity. Lim: Expensive instrumentation, complex sample preparation.	[24]
Spectrofluorimetry (Carbon Quantum Dots)	-	0.01 µg/mL	Dairy products	Adv: High selectivity. Lim: Time-consuming nanoprobe synthesis.	[25]
Spectrofluorimetry (Silver nanoparticles nanoprobe)	-	0.0256 µg/mL	Environmental, Biological, Pharmaceutical	Adv: High selectivity. Lim: Complex probe design and fabrication.	[26]
HILIC-HPLC-MS/MS	-	0.000006 µg/mL	Water	Adv: Extreme sensitivity for trace analysis. Lim: Very high instrument cost, requires expert operation.	[23]

The novelty of the proposed method is its optimal balance of high sensitivity and exceptional practicality, positioning it as a superior alternative for routine analysis compared to recent, more complex fluorometric techniques. In addition to its high sensitivity, the proposed procedure is rapid, requires only a single reaction step with one reagent, and is performed at room temperature. This stands in stark contrast to the complex sample preparation, lengthy analysis times, or sophisticated instrumentation required by the methods listed in Table 2. Although fluorescamine is relatively costly, the enhanced sensitivity, simplicity, and speed of the overall method provide a distinct advantage for the routine analysis of streptomycin in pharmaceutical and clinical laboratories.

#### Accuracy and precision

Accuracy of the proposed method was evaluated using five streptomycin concentrations ranging from 100 to 600 ng/mL were analyzed, each in triplicate. The results, expressed as % recovery ± SD, are summarized in Table S1. The mean recovery was 99.69 ± 0.83%, demonstrating excellent accuracy. Method precision was further examined at two levels: intra-day and inter-day reproducibility. Streptomycin solutions at 250, 350, and 450 ng/mL were analyzed in triplicate within a single day and over three consecutive days. The RSD values obtained were all below 2% (Table S2), indicated that the suggested approach has a high level of repeatability.

#### Robustness

Robustness was evaluated by introducing minor variations in experimental parameters (pH, buffer volume, and fluorescamine concentration) during the analytical procedure. These modifications did not significantly affect fluorescence intensity, confirming its robustness (Table S3).

## Applications

### Streptomycin assay in vials

Following complete validation, the developed analytical method was successfully applied to the determination of streptomycin in its commercial vial formulation. The results obtained were statistically compared with those of a reported method [15]. At the 95% confidence level, both the Student’s *t*-test and *F*-test revealed no significant differences between the two methods, as the calculated values did not exceed the corresponding theoretical ones. This gives an idea of the suggested method acceptable level of accuracy (Table 3).

**Table 3:** Application of the proposed spectrofluorimetric method for assay of streptomycin in strep dosage form

Parameter	streptomycin^®^ injection	
	Proposed method	Reported method [15]
% Recovery	99.69	100.2
Standard deviation (SD)	0.83	0.6
Number of determinations	5	5
t-Value^a^	1.02	
f-Value^a^	3.6	

^a^ tabulated value at 95% confidence limit; t = 2.306 and F = 6.338

### Streptomycin assay in human plasma

The proposed method could be used to successfully detect Streptomycin in spiked plasma. Streptomycin was absorbed quickly, and maximum drug concentrations (C_max_) in plasma was reached approximately 1 h after intramuscular administration of 1000 mg strep. The mean C_max_ values was 25 –20 µg/mL (which is equivalent to 25,000–50,000 ng/mL) [31, 32]. Standard solutions of Streptomycin (100, 200, 300, 400, and 500) ng/mL were added to 1mL drug-free plasma, mixed, and analyzed as stated in Experimental Sect. 2.6. For each concentration, three independent measurements were made. In spiked human plasma samples, the percentage recovery of streptomycin ranged from 98.1% to 101.3%, with relative standard deviation (RSD) values between 0.3% and 1.6% (Table 4), confirming the accuracy and precision of the method in biological matrices.

**Table 4:** Application of the proposed spectrofluorimetric method for assay of streptomycin in spiked human plasma

Parameter	Added Conc. (ng/mL)	Found^a^ (ng/mL)	% Recovery ± SD
Spiked human plasma	100	98.116	98.11 ± 1.4
	200	202.621	101.3 ± 0.32
	300	297.96	99.3 ± 1.6
	400	396.63	99.1 ± 0.51
	500	493.02	98.6± 1.4

^a^ Mean of five determinations, SD: standard deviation

To ensure the reliability of the method for handling real clinical samples, the stability of streptomycin in spiked human plasma was evaluated under typical storage and handling conditions. Short-term stability was assessed by keeping spiked samples at ambient temperature (25 °C) for 6 h prior to processing and analysis. Freeze-thaw stability was evaluated by subjecting aliquots to three complete cycles between − 20 °C and 25 °C. As summarized in Table S4, the measured concentrations showed excellent recovery (99.7% for short-term; 99.5% after three freeze-thaw cycles) with low RSD values (< 2%), confirming that streptomycin remains stable in plasma under these conditions. These findings confirm that streptomycin is stable in plasma under typical handling and storage conditions, ensuring the reliability of the proposed method for real clinical or pharmacokinetic applications.

### Evaluation of possible matrix interferences

Although the present work used spiked human plasma samples, potential matrix effects were considered. The protein precipitation step with acetonitrile effectively removed most plasma proteins, as indicated by the absence of background fluorescence in blank plasma samples processed under identical conditions. The fluorescence spectrum of the blank extract showed no signal at 482 nm, confirming negligible endogenous interference. Furthermore, recoveries between 98.1% and 101.3% with RSD values below 1.6% indicate that the matrix did not significantly influence the analytical response.

Endogenous metabolites such as amino acids and peptides can, in principle, react with fluorescamine due to their primary amino groups. However, their contribution to background fluorescence is minimized by the large dilution factor (100 µL plasma in 3 mL acetonitrile + subsequent dilution) and by measuring at a wavelength where the streptomycin–fluorescamine adduct exhibits a distinct excitation/emission profile (390/482 nm).

Nevertheless, for spiked and quality-control samples the current method demonstrates adequate selectivity and robustness, making it suitable for rapid routine monitoring and formulation testing.

## Conclusion

The present work describes the development and validation of a straightforward, fast, and robust spectrofluorimetric method for the quantification of streptomycin in bulk material, pharmaceutical formulations, and spiked human plasma. The reaction took place in a single pot at room temperature. As a result, the proposed method is more advantageous than the previously described methods, which relied on heating the drug with the reagent at high temperatures for a lengthy period of time. Furthermore, unlike chromatography and electrochemical approaches, the method used is inexpensive and simple. As a consequence of its enhanced simplicity, superior sensitivity, and independence from sophisticated or expensive equipment, the proposed method can be used in Streptomycin quality control analysis. Moreover, the proposed method was successfully applied in a biological fluid sample.

## Supplementary Information

Below is the link to the electronic supplementary material.

Supplementary Material 1. Fig. S1. The chemical structure of Streptomycin. Table S1: Evaluation of the accuracy of the proposed spectrofluorimetric method of Streptomycin in pure form. Table S2: Precision data for the determination of Streptomycin in pure form by the proposed spectrofluorimetric method. Table S3: Evaluation of the robustness of the proposed method. Table S4: Stability of streptomycin in spiked human plasma under typical storage and handling conditions (n = 3).

## Data Availability

The datasets generated and/or analyzed during the current study are available from the corresponding author upon reasonable request.

## Abbreviations

Strep.: Streptomycin
Fl: Fluorescence intensity
LOD: Limit of detection
LOQ: Limit of quantitation
RSD: Relative standard deviation
HPLC: High-performance liquid chromatography
LC-MS/MS: Liquid chromatography–tandem mass spectrometry
