# Supplementary material for: Turning on fluorescent probe for sensitive detection of streptomycin in pure, pharmaceutical formulations and human plasma
Source: BMC Chem. 2026 Jan 17;20(1):25. doi: 10.1186/s13065-025-01708-7 (PMC12896356; doi:10.1186/s13065-025-01708-7)
Supplement: Supplementary file 1 — Supplementary Material 1. Fig. S1. The chemical structure of Streptomycin. Table S1: Evaluation of the accuracy of the proposed spectrofluorimetric method of Streptomycin in pure form. Table S2: Precision data for the determination of Streptomycin in pure form by the proposed spectrofluorimetric method. Table S3: Evaluation of the robustness of the proposed method. Table S4: Stability of streptomycin in spiked human plasma under typical storage and handling conditions (n = 3). [file 13065_2025_1708_MOESM1_ESM.docx]

**Turning on fluorescent probe for sensitive detection of streptomycin in pure, pharmaceutical formulations and human plasma**

**Bassant Samy ^1^ ,Mokhtar M. Mabrouk ^2,3^, Mohamed A. Abdel Hamid ^2,3^, Hytham M. Ahmed ^1,4^**

^1^Pharmaceutical Analysis Department, Faculty of Pharmacy, Menoufia University, Shebin Elkom, Menoufia, Egypt.

^2^Department of Pharmaceutical Analytical Chemistry, Faculty of Pharmacy, Tanta University, Tanta, El Gharbeia, Egypt.

^3^Department of Pharmaceutical Chemistry, Faculty of Pharmacy, Alsalam University, Kafr El Zayat, El Gharbeia, Egypt.

^4^Pharmaceutical Analytical Chemistry Department, Faculty of Pharmacy, Menoufia National University, 70 km Cairo-Alexandria agricultural road, Menoufia, Egypt.

Corresponding author: Tel: 00201004844589

E-mail: [hmaahmed@yahoo.co.uk](mailto:hmaahmed@yahoo.co.uk)


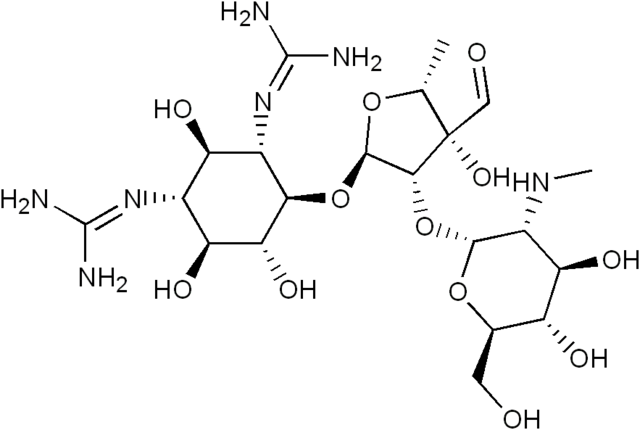


**Fig. S1:** The chemical structure of Streptomycin.

**Table S1:** Evaluation of the accuracy of the proposed spectrofluorimetric method of Streptomycin in pure form.

| Sample number | Conc taken(ng/mL) | Found ^a^ (ng/mL) | % Recovery ± SD |
| --- | --- | --- | --- |
| 1 | 150 | 150.12 | 100.08± 0.52 |
| 2 | 250 | 248.133 | 99.3±0.66 |
| 3 | 350 | 348.85 | 99.6±0.315 |
| 4 | 450 | 453.14 | 100.6±0.2 |
| 5 | 550 | 547.85 | 99.6±0.46 |

**^a^** Mean of three replicate measurements, SD: standard deviation.

**Table S2:** Precision data for the determination of Streptomycin in pure form by the proposed spectrofluorimetric method.

| Precision level | Conc. taken  (ng/mL) | Found ^a^  (ng/mL) | % Recovery ± RSD |
| --- | --- | --- | --- |
|  | 250 | 249.9 | 99.9±0.1.23 |
| Intra-day | 350 | 350.3519 | 100.2±01.7 |
|  | 450 | 452.303 | 100.5±1.1 |
|  |  |  |  |
|  | 250 | 249.1 | 99.6±0.34 |
| Inter-day | 350 | 349.1 | 99.7±0.82 |
|  | 450 | 451.17 | 100.26±.67 |

**^a^** Mean of three replicate measurements, RSD: relative standard deviation

**Table S3**: Evaluation of the robustness of the proposed method.

| Variables | Value | % Recovery ± SD ^a^ | |
| --- | --- | --- | --- |
| Optimum 100.3 ± .38  (PH=8 , 1ml borate buffer, 0.75 ml fluorescamine solution) | | | |
| PH | PH 7.8  pH 8.2 | | \| 99.5±1.03 \| \| --- \| \| 99.8±0.95 \| |
| Borate buffer volume | 0.8ml  1.2ml | | \| 100.4±0.87 \| \| --- \| \| 100.5±1.3 \| |
| Fluorescamine volume | 0.7 ml  0.8 ml | | \| 100.2±0.67 \| \| --- \| \| 99.6±0.98 \| |

^a^ the average of three determination

**Table S4:**  Stability of streptomycin in spiked human plasma under typical storage and handling conditions (n = 3).

| Stability Condition | Nominal Conc. (ng/mL) | Measured Conc. (ng/mL, mean ± SD, n=3) | % Recovery | RSD (%) | Comment |
| --- | --- | --- | --- | --- | --- |
| Short-term (6 h, 25 °C) | 300 | 299.2 ± 3.1 | 99.7 | 1.0 | Stable |
| Freeze–thaw (3 cycles, −20 °C ↔ 25 °C) | 300 | 298.5 ± 3.8 | 99.5 | 1.3 | Stable |
